# Study Protocol for Radiation Exposure and Cancer Risk Assessment: The Taiwan Nuclear Power Plants and Epidemiology Cohort Study (TNPECS)

**DOI:** 10.2188/jea.JE20210020

**Published:** 2023-01-05

**Authors:** Wei-Te Wu, Cheng-Ya Pan, Szu-Li Chang, Yi-Hau Chen, Chuan-Jong Tung, Pinpin Lin

**Affiliations:** 1 National Institute of Environmental Health Sciences, National Health Research Institutes, Zhunan, Taiwan; 2 Institute of Environmental and Occupational Health Sciences, National Yang Ming University, Taipei, Taiwan; 3 Medical Physics Research Center, Institute for Radiological Research, Chang Gung University, Taoyuan, Taiwan; 4 Institute of Nuclear Engineering and Science, National Tsing Hua University, Hsinchu, Taiwan; 5 Radiation Protection Association, Republic of China (ROC), Hsinchu, Taiwan; 6 Institute of Statistical Science, Academia Sinica, Taipei, Taiwan; 7 Department of Medical Imaging and Radiological Sciences, College of Medicine, Chang Gung University, Taoyuan, Taiwan

**Keywords:** cohort study, radiation, nuclear power plants, cancer risk assessment

## Abstract

**Background:**

This cohort was established to evaluate whether 38-year radiation exposure (since the start of nuclear reactor operations) is related to cancer risk in residents near three nuclear power plants (NPPs).

**Methods:**

This cohort study enrolled all residents who lived within 8 km of any of the three NPPs in Taiwan from 1978 to 2016 (*n* = 214,502; person-years = 4,660,189). The control population (*n* = 257,475; person-years = 6,282,390) from three towns comprised all residents having lived more than 15 km from all three NPPs. Radiation exposure will be assessed via computer programs GASPAR-II and LADTAP-II by following methodologies provided in the United States Nuclear Regulatory Commission regulatory guides. We calculated the cumulative individual tissue organ equivalent dose and cumulative effective dose for each resident. This study presents the number of new cancer cases and prevalence in the residence-nearest NPP group and control group in the 38-year research observation period.

**Conclusion:**

TNPECS provides a valuable platform for research and opens unique possibilities for testing whether radiation exposure since the start of operations of nuclear reactors will affect health across the life course. The release of radioactive nuclear species caused by the operation of NPPs caused residents to have an effective dose between 10^−7^ and 10^−3^ mSv/year. The mean cumulative medical radiation exposure dose between the residence-nearest NPP group and the control group was not different (7.69; standard deviation, 18.39 mSv and 7.61; standard deviation, 19.17 mSv; *P* = 0.114).

## INTRODUCTION

Epidemiologic studies of cancer risks in residents who live near nuclear power plants (NPPs) have been carried out in at least eleven countries (South Korea, Canada, Finland, France, Germany, Great Britain, Japan, Spain, Sweden, Switzerland, and the United States).^1^^–^^16^ The majority of these studies investigated rates of cancer deaths or cancer incidence in residents living in various-distance geographic units, including counties and municipalities. These studies have come to different conclusions, with some suggesting a positive association between living in proximity to a nuclear facility and cancer risks. However, most of these studies did not present information about the radiation dose level in residents, and used only a surrogate, such as residence distance from NPPs. They assumed that the farther one resides from a NPP, the lower the dose one might receive; however, this might not always hold true.

Several epidemiologic studies (eg, the German KiKK study and the French Geocap study) have shown higher than expected incidence rates of acute leukemia in children living near NPPs,^9^^,^^16^^–^^20^ but there have been no reports of clustering of childhood leukemia near NPPs after 2008 in other studies.^19^ These results have helped rekindle the debate about the health effects of environmental exposure to these plants under their normal operating conditions. Therefore, researchers began to discuss that these positive findings may have limitations in the research design, such as (1) use of the distance between the place of residence and the NPP, which may lead to misclassification of radiation exposure; (2) the absence of systematic and long-term cancer follow-up data; and (3) poor control for confounding factors.

In 2011, the Great East Japan Earthquake and subsequent tsunami hit the Fukushima Daiichi NPP, causing it to release radioactive elements. This nuclear accident renewed public concern throughout the world, in particular in nearby Taiwan, even though it is very different to the situation with normal operation. Taiwan has three NPPs (including 6 nuclear reactors), and Taiwan is the seventeenth most densely populated country in the world, with approximately 651 people per square kilometer. A quarter of a million people live within an 8-km radius of one of the three NPPs. Taiwan has had six epidemiological studies of health effects in residents living near NPPs.^21^^–^^23^ However, these studies have lacked systematic surveillance of long-term follow-up health effects and detailed individual radiation exposure assessments in relation to the NPPs. Moreover, the theory of linear no-threshold (LNT) controversy is whether there is a threshold (low dose limit or threshold) for radiation-sensitive cancers. Although a linear non-threshold model can give an estimate at low dose, it is just for principle of maximum protection for individuals. The related scientific evidence has not been recognized. This study can provide data for assessing cancer risk at low dose.

This cohort was established to evaluate whether 38-year radiation exposure since the start of operation of the nuclear reactors is related to cancer risk in residents near any of the three NPPs. The objective of this paper is to introduce the study profile and protocol in The Taiwan Nuclear Power Plants and Epidemiology Cohort Study (TNPECS) for improving methodology of exposure assessment. The hypothesis of this study is that exposed to low-dose ionizing radiation produced by NPPs can elevate cancer risks.

## METHODS

### Study design and procedures

**Figure 1.**
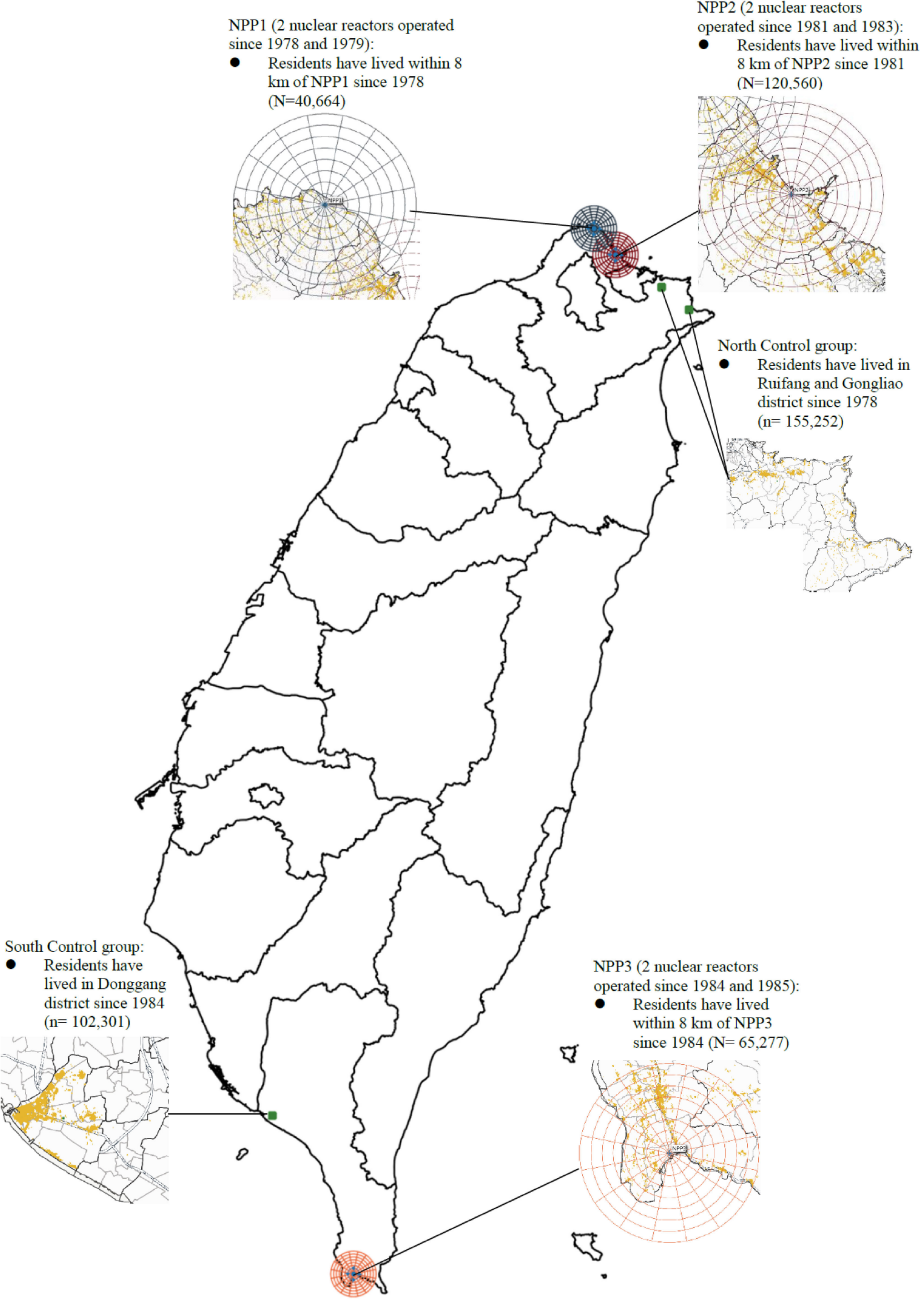
Study map and population distribution in an 8-km radius of three Taiwan nuclear power plants (NPPs). Note: Human migration toward or away from the three NPPs occurred during the 38-year period. Therefore, the note in this figure shows the numbers of ever-stayed or residents around 3 NPPs.

Taiwan has 3 NPPs, with 6 nuclear reactors operating since 1978 (Figure 1). The Taiwan Nuclear Power Plants and Epidemiology Cohort (TNPPEC) study is a long-term cohort study aimed at evaluating cancer risks in people within an 8-km radius of three Taiwan NPPs. This study applied the detailed migration household registration records (1,209,009 person-times) from the Ministry of the Interior. Registration records included all moving-in registration, such as births, marriage, residential change in, original residents, and migration in, and all moving-out registration, such as death, divorce, residential change out, and migration out. Therefore, we can effectively grasp the migration change of each individual in the research area. After we used national ID numbers to identify individuals, the total number was 471,977, and then detailed geographic information system (GIS) positioning was performed. The coordinate reference system EPSG: 3826 (TWD97/121 subzone) was used to locate the household-registration data (geocoding) through the national house address location service of the geographic information map cloud service platform built by the Information Center of the Ministry of the Interior, Taiwan. We used the Quantum GIS 3.2.0 (QGIS Association, http://www.qgis.org) software package for GIS spatial distribution mapping.

The cohort population was established by household registration to retrieve all residents having stayed near (within 8 km) of any of the three NPPs in Taiwan (Figure 1), and it was conducted between January 1978 and December 2016 to trace when these residents first moved in and moved out. Based on 214,502 persons and 4,660,189 person-years of follow-up data, the mean ages at the first year of residency and years of residence in total residence-nearest NPP population were 22.6 (standard deviation [SD], 20.7) and 15.0 (SD, 8.8) years, respectively (Table 1). About 52.2% of the participants were female, and most of the participants lived within 2 to 6 km of the NPPs (70.8%). About 11.6% of them died between 1985 and 2016. Age at first residency for each period is presented in eTable 1.

**Table 1.** Demographic characteristics of residents who have lived within 8 km of NPPs and residents who have lived in control districts

	Total residence-nearest NPP population (*n* = 214,502)		Residence-nearest NPP1 group (*n* = 40,664)^b^		Residence-nearest NPP2 group (*n* = 120,560)^b^		Residence-nearest NPP3 group (*n* = 65,277)^b^		Total controls (*n* = 257,475)		North control group (*n* = 155,252)^b^		South control group (*n* = 102,301)^b^	
	*mean*	*SD*	*mean*	*SD*	*mean*	*SD*	*mean*	*SD*	*mean*	*SD*	*mean*	*SD*	*mean*	*SD*
Age in the first of residency, years	22.6	20.7	21.2	20.6	23.0	21.1	22.4	20	20.7	19.4	20.2	19.5	21.5	19.2
Years of residence^a^	15.0	8.8	17.3	9.3	15.2	9.3	14.1	7.8	16.1	8.5	17.5	8.5	14.1	8.2
	*n*	*%*	*n*	*%*	*n*	*%*	*n*	*%*	*n*	*%*	*n*	*%*	*n*	*%*
Gender														
Male	102,510	47.8	19,450	47.8	57,917	48.0	30,746	47.1	125,079	48.6	75,905	48.9	49,211	48.1
Female	111,992	52.2	21,214	52.2	62,643	52.0	34,531	52.9	132,388	51.4	79,342	51.1	53,087	51.9
Missing	0		0		0		0		8		5		3	
Initial residency period														
1978–1982	55,757	26.0	20,732	51.0	41,371	34.3	98	0.2	94,426	36.7	94,426	60.8	49	0.1
1983–1987	49,052	22.8	3,406	8.4	10,013	8.3	36,756	56.3	57,483	22.3	9,303	6.0	48,190	47.1
1988–2000	49,063	22.9	7,680	18.9	32,399	26.9	11,144	17.0	46,916	18.2	24,706	15.9	22,226	21.7
2001–2015	60,630	28.3	8,846	21.7	36,777	30.5	17,279	26.5	58,650	22.8	26,817	17.3	31,836	31.1
Distance from NPPs														
≤1.0 km	616	0.3	264	0.6	163	0.1	220	0.3	0	0	0	0	0	0
1.1–2.0 km	13,132	6.1	2,335	5.7	2,965	2.5	7,989	12.2	0	0	0	0	0	0
2.1–3.0 km	30,124	14.0	8,848	21.8	18,383	15.2	3,472	5.3	0	0	0	0	0	0
3.1–4.0 km	56,659	26.4	3,280	8.1	46,621	38.7	7,399	11.3	0	0	0	0	0	0
4.1–5.0 km	26,860	12.5	10,308	25.3	7,492	6.2	12,540	19.2	0	0	0	0	0	0
5.1–6.0 km	38,499	17.9	8,658	21.3	13,753	11.4	21,359	32.7	0	0	0	0	0	0
6.1–7.0 km	17,093	8.0	4,392	10.8	8,790	7.3	5,600	8.6	0	0	0	0	0	0
7.1–8.0 km	31,519	14.7	2,579	6.3	22,393	18.6	6,698	10.3	0	0	0	0	0	0
>15 km	0	0.0	0	0	0	0	0	0	257,475	100.0	155,252	100.0	102,301	100.0
Salary grades for National Health Insurance (NT$)														
<660	42,250	19.7	5,979	14.7	21,524	17.9	16,442	25.2	45,169	17.5	29,273	18.9	15,922	15.6
660–729	20,440	9.5	3,740	9.2	10,506	8.7	7,226	11.1	24,825	9.6	13,964	9.0	10,873	10.6
730–799	60,704	28.3	13,708	33.7	34,590	28.7	16,663	25.5	69,925	27.2	34,639	22.3	35,301	34.5
800–1,119	33,346	15.5	5,907	14.5	19,380	16.1	9,797	15.0	41,031	15.9	26,473	17.1	14,570	14.2
1,200–1,500	23,053	10.7	4,455	11.0	13,959	11.6	5,953	9.1	30,184	11.7	20,236	13.0	9,953	9.7
>1,500	21,777	10.2	3,700	9.1	13,405	11.1	5,674	8.7	28,967	11.3	18,410	11.9	10,564	10.3
Missing	12,932		3,175		7,196		3,522		17,374		12,257		5,118	
Death status between 1985–2016														
Alive	189,530	88.4	35,621	87.6	107,583	89.2	56,807	87.0	224,584	87.2	133,410	85.9	91,247	89.2
Dead	24,972	11.6	5,043	12.4	12,977	10.8	8,470	13.0	32,891	12.8	21,842	14.1	11,054	10.8

NPP, Nuclear Power Plant.

^a^Sum of residence time of different household registration tracking until cancer or death or study ends.

^b^Human migration around three NPPs occurred during the 38-year period. Therefore, the note in table shows the numbers of ever-stayed or resident near 3 NPPs or within control area.

Moreover, the control population was established by household registration with the same urban characteristics and scale as the cohort population. This study retrieved all residents having stayed far (more than 15 km) from any of the three NPPs in Taiwan from three towns (Ruifang Town, Gongliao Town, and Donggang Town) (Figure 1), and it was conducted between January 1978 and December 2016 to trace when these residents first moved in and moved out. This was based on 257,475 persons and 6,282,390 person-years of follow-up data. This study adopted Luo’s research (1992) to select control townships of appropriate comparability with NPPs townships, which used 2000 Taiwan census data to establish the development stratification of Taiwan townships. This paper considered variables including population density (people/km^2^), the population ratio of people with college or above educational levels, the population ratio of people over 65 years old, the population ratio of agriculture workers, and the number of physicians per 100,000 people. It also used cluster analysis, with squared Euclidean distance and Wald’s minimum variance method, to study the urbanization stratification; the results identified seven clusters. Therefore, this study confirms that regional characteristics were similar between those living near NPPs and the control group.

This study was approved by the Institutional Review Board of the National Health Research Institutes, Taiwan (NIRB file number EC1051106). The constitution and operation of the review board are formulated according to the guidelines of International Conference on Harmonization-Good Clinical Practice (ICH-GCP). The authors confirm that all experiments were performed in accordance with relevant guidelines and regulations. Two project advisory boards were convened for peer-review twice a year: an academic advisory committee, for assessing the reliability and validity of the research, and a policy translation advisory committee, for communicating with local governments and relevant agencies. Academic advisors invited with professional experience from domestic academic institutions, including health physicists, environmental and occupational disease specialists, epidemiologists, biomedical statistics experts, and oncologists. Policy translation advisors invited with the following units to recommend representatives: (1) relevant authorities, including the Ministry of Economy, Ministry of Health and Welfare, and Atomic Energy Commission of Executive Yuan; (2) local government where nuclear power plant is located, including New Taipei City Government and Pingtung County Government; (3) scholars and experts (independently invited by the investigation team); and (4) The Taiwan Power Company. The members of the advisory committee had no role in the decision to publish, or preparation of this manuscript.

### Data collection

**Figure 2.**
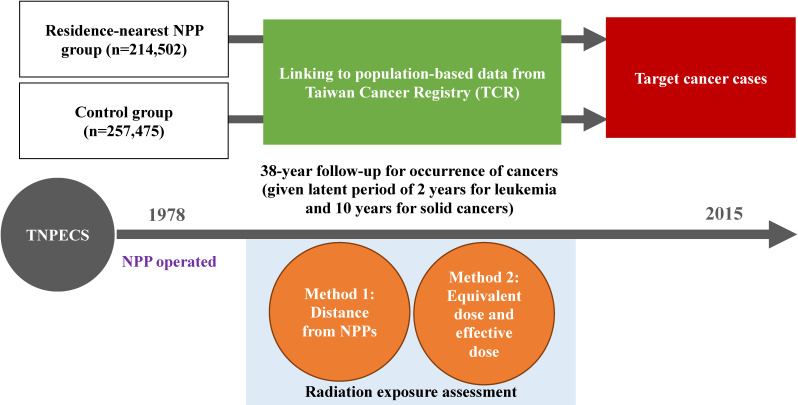
Flowchart of Taiwan Nuclear Power Plants and Epidemiology Cohort (TNPPEC) study.

This cohort was established to evaluate whether 38 years of radiation exposure is related to cancer risk in residents who live near three NPPs. The cohort and control population were linked to the Taiwan Cancer Registry (TCR) to follow up the 38 years of cancer incidence information (Figure 2). The target cancers were based on the International Classification of Disease for Oncology 3rd edition (ICD-O-3) and the 2008 World Health Organization classification of lymphoid neoplasms and beyond (Table 2). The selection of target cancers was based on international organizations identified or mentioned cancer sites related to ionizing radiation (eTable 2). Cases with a behavior code of 2 (in situ) or 3 (malignant) in the ICD-O-3 were included in the registry. Meanwhile, the histology (morphology) of the malignancies was identified according to the ICD-O-3.

**Table 2.** Target outcomes based on the ICD-O-3 and the 2008 WHO classification of lymphoid neoplasms

Sites/Types	2008 WHO Classification/ICD-O-3 Codes
Overall Cancer	C00–C80
1. Malignant neoplasm of lymphatic and haemopoietic tissue	
Lymphocytic leukemia	M-98003–M-99643, M-99803–M-99893
Leukemia (excluded Chronic lymphocytic)	
Lymphoma	C77(M8000–M9589)
Hodgkin’s lymphoma	M-96503–M-96673 (96503/96513/96523/96533/ 96593/96613/96623/96633/96643/96653/96673)
Non-Hodgkin’s lymphoma	M-95903–M-95963, M-96703–M-97293, M-97503–M-97583, M-97643
Multiple myeloma	M-9731–M-9732, M9734
2. Malignant neoplasm of lip, oral cavity and pharynx	
Oral	C00–C06
Oral cavity and pharynx	C00–C14
Major salivary glands	C07–C08
3. Malignant neoplasm of digestive organs and peritoneum	
Esophagus	C15
Stomach	C16
Colon (excluding rectum)	C18
Rectum and rectosigmoid junction, sigmoid colon, anus	C19–C21
Rectum and rectosigmoid junction	C19–C20
Sigmoid colon	C187
Anus, anal canal, and anorectum	C21
Liver and intrahepatic bile duct	C22
4. Malignant neoplasm of respiratory and intrathoracic organs	
Lung	C34
Trachea and bronchi	C339, C340
5. Malignant neoplasm of bone, connective tissue, skin and breast	
Bones, joints, and articular cartilage	C40–C41
Connective, subcutaneous and other soft tissues	C47, C49
Melanoma of skin	C44 (M-87203–M-87903)
Other non-melanoma skin cancer, excluding basal and squamous cell carcinoma	C44 (excluding M-87203–M-87903)
Female breast	C50
6. Malignant Neoplasm of Genital Organs	
Uterus, not otherwise specified	C55
Ovary, fallopian tube, and broad ligament	C56, C570–C574
Prostate gland	C61
7. Malignant neoplasms of urinary tract	
Bladder	C67
Kidney	C64
8. Malignant neoplasm of nervous system	
Brain	C71
Other and unspecified parts of nervous system	C70, C72
9. Thyroid gland	C73

ICD-O-3, International Classification of Diseases for Oncology.

Researchers used national ID numbers to identify new cancer cases and information about cancer among the cohort and control population from the TCR from January 1, 1978, to December 31, 2015. This study excluded subjects who has received thermoluminescent dosimeters for NPP workers (*n* = 12,195) including regular, contract, temporary, and casual workers, and subjects who had a cancer diagnosis before moving into the area (*n* = 1,560). Moreover, the analysis of cancer occurrences all consider the incubation period of radiation exposure to cancer. This study uses the hypothesis announced in the 2017 report of the United Nations Scientific Committee on the Effects of Atomic Radiation. The incubation period for leukemia is 2 years and for other cancers is 10 years.^24^ This study excluded residents with newly diagnosed solid tumors who had not lived in the area for more than 10 years, and residents with newly blood cancer who had lived near the NPPs for less than 2 years.

The cohort and control population were linked to the Taiwan Death Register, and the National Health Insurance Database to follow up the 38 years of death and medical records.

### Exposure assessment

#### Input data for radiation exposure dose evaluation

Although distance is often used an alternative indicator of radiation exposure, it does not represent the level of exposure dose. Potential radiation sources include air and aquatic radioactive effluent discharged from NPPs, and the exposure pathways may be contributed externally, such as through atmospheric dispersion, or internally, such as through consuming crops grown in the area where radioactive particles might deposit. Only if one considers exposure pathways comprehensively and gathers statistical data for dose calculation completely will the accuracy of the dose level be guaranteed. Therefore, the estimation of the actual dose level one receives is still a necessary index for one to study the effects of long-term low-dose radiation on health.

Radiation exposure will be assessed using computer programs GASPAR-II and LADTAP-II (Chesapeake Nuclear Services, Inc., Annapolis, Maryland) following methodologies provided in the United States Nuclear Regulatory Commission regulatory guides.^25^ These will assess the radiological impact of routine discharges of radioactive material into the environment. The radiation emissions in air, water, and food will be inputted to calculate the internal and external doses. The exposure pathways can distinguish the contribution of airborne (GASPAR-II) and waterborne (LADTAP-II) means. Some pathways, including plume, ground, and inhalation, vegetable, meat, milk, and goat milk, are calculated by GASPAR-II, while the rest are calculated by LADTAP-II (Table 3). The radiation dose in resident near NPPs assess from airborne and waterborne effluent releases. The dose affect factors of airborne effluent include air submersion, inhalation, ingestion (crops and livestock), and external irradiation. The dose affect factors of waterborne effluent include immersion (water sports), ingestion (aquatic products), and external irradiation from beach activities.

**Table 3.** Parameters to determine the individual radiation equivalent dose and effective dose

Parameter		Range of the parameters
Number of NPPs		1 (Jinshan, Ground-Level Release), 1′ (Jinshan, Stack Release), 2 (Guosheng, Ground-Level Release), 3 (Ma’anshan, Ground-Level Release)

Year		1978∼2015

Age categories		Infant-3 (3-month-old infant), Child-1 (1-year-old child), Child-5 (5-year-old child), Child-10 (10-year-old teenager), Teen-15 (15-year-old teenager) and Adults

Geographical location	Orientation (Angular)	N, NNE, NE, ENE, E, ESE, SE, SSE, S, SSW, SW, WSW, W, WNW, NW, NNW

	Distance (Radial)	0.8 km, 1.25 km∼7.75 km (0.5 km per step)

Exposure pathway (Airborne)^a^	External	Submersion (Plume) External irradiation (Ground)

	Internal	Inhalation (Air) Ingestion (Crops livestock)

Exposure pathway (Waterborne)^b^	External	External irradiation (Shoreline activity)

	Internal	Ingestion (Aquatic products) Immersion (Boating, Swimming)

Dose conversion factors	External	Airborne (IAEA 115, FGR 13); Waterborne (DCFPAK ORNL/TM-13347, DOE/EH-0070)

	Internal	Airborne (ICRP 72); Waterborne (ICRP 72)

Organ/Effective Dose	Organ	Equivalent dose (adrenals, bladder wall, bone surface, brain, breast, esophagus, stomach wall, small intestine wall, upper large intestine wall, lower large intestine wall, kidney, liver, muscle, ovaries, pancreas, red marrow, lungs, skin, spleen, testes, thymus, thyroid, uterus, air way, colon, gallbladder wall, heart wall.)

	Effective	Effective dose

IAEA, International Atomic Energy Agency; ICRP, International Commission on Radiological Protection; FGR 13, Federal Guidance Report No. 13, U.S. Environmental Protection Agency; DCFPARK, Dose coefficient data file package for Sandia National Laboratory (DCFPAK); DOE, U.S. Department of Energy.

^a^Computer programs GASPAR-II by following methodologies provided in USNRC Regulatory Guides (NRC, 1977a).

^b^Computer programs LADTAP-II by following methodologies provided in USNRC Regulatory Guides (NRC, 1977a).

According to the above radiation-exposure assessment method, this study exported the radiation-exposure dose datasets by over with 16 distances, 16 orientation for the NPPs from the start of their official operation until 2016. More detailed information for assessment of resident doses near NPPs in Taiwan was presented in another paper.^26^ Then we used dose conversion factors with external (Airborne: IAEA 115, FGR 13; Waterborne: DCFPAK ORNL/TM-13347, DOE/EH-0070) and internal (Airborne [ICRP 72]; Waterborne [ICRP 72]) exposures to establish a detailed database of organ-equivalent and effective doses (Table 3). The annual and age-category equivalent dose of various organs and the effective dose were constructed using 16 angular segments, and 15 radial segments subdivide the areas surrounding the three NPPs within a range of 8 km.

#### Cumulative dose of personal radiation

According to the age group, NPP number, distance and orientation, actual residency period, and the proportion of each residency year for each resident, we calculated the cumulative individual tissue organ equivalent dose and cumulative effective dose. The calculation formula is as follows:

$$TD=\sum_{\text{T}=\text{ts}}^{\text{te}}f^{\text{T}}D_{\text{a}}^{\text{T}}(X,r,\theta )$$

TD: total individual radiation equivalent dose or effective dose
T: years of residence
t_s_: each residency starting year
t_e_: each residency ending year
a: age category
f^T^: percentage of residence at residency year (%)
D_a_^T^: yearly radiation equivalent dose or effective dose each age category (mSv)
X: nearest NPP
r: distance of residence from the nearest NPP
θ: orientation of residence from the nearest NPP

The release of radioactive nuclear species caused by the operation of NPPs resulted in residents having an effective dose between 10^−7^ and 10^−3^ mSv/year. Table 4 presents the quartiles of the cumulative equivalent dose and cumulative effective dose of individual tissues and organs for each resident. To assess the difference between the two methods and to compare with previous studies, this study adopted two radiation exposures assessments including (1) distance from NPPs and (2) equivalent dose and effective dose (Figure 2).

**Table 4.** Distribution of cumulative radiation effective dose and equivalent dose among residence-nearest NPP group (*n* = 214,502)

	Lower quartile (mSv)	Median (mSv)	Upper quartile (mSv)
Effective dose	6.26 × 10^−5^	1.792 × 10^−4^	1.973 × 10^−3^
(Organ) Equivalent dose			
Red marrow	6.57 × 10^−5^	1.955 × 10^−4^	1.425 × 10^−3^
Brain	5.56 × 10^−5^	1.575 × 10^−4^	1.279 × 10^−3^
Esophagus	5.08 × 10^−5^	1.436 × 10^−4^	1.212 × 10^−3^
Stomach wall	5.43 × 10^−5^	1.532 × 10^−4^	1.267 × 10^−3^
Colon	3.39 × 10^−5^	1.145 × 10^−4^	0.802 × 10^−3^
Lower large intestine wall	6.41 × 10^−5^	1.983 × 10^−4^	1.859 × 10^−3^
Liver	6.24 × 10^−5^	1.876 × 10^−4^	1.519 × 10^−3^
Lungs	5.57 × 10^−5^	1.572 × 10^−4^	1.396 × 10^−3^
Airway	2.62 × 10^−5^	7.98 × 10^−5^	6.165 × 10^−4^
Bone surface	8.28 × 10^−5^	2.371 × 10^−4^	1.834 × 10^−3^
Skin	7.71 × 10^−5^	2.191 × 10^−4^	2.359 × 10^−3^
Breast	5.77 × 10^−5^	1.628 × 10^−4^	1.305 × 10^−3^
Uterus	5.24 × 10^−5^	1.490 × 10^−4^	1.296 × 10^−3^
Ovaries	5.53 × 10^−5^	1.576 × 10^−4^	1.360 × 10^−3^
Testes	5.58 × 10^−5^	1.569 × 10^−4^	1.335 × 10^−3^
Bladder wall	5.30 × 10^−5^	1.489 × 10^−4^	1.576 × 10^−3^
Kidney	5.43 × 10^−5^	1.532 × 10^−4^	1.272 × 10^−3^
Thyroid	7.45 × 10^−5^	2.335 × 10^−4^	2.884 × 10^−3^

^*^NPP, Nuclear Power Plant.

### Statistical analyses

Participants who survived and did not have cancers before the cutoff date (December 31, 2015) contributed to the person-year time between the date on the first of residency and cutoff date. Participants who survived and had cancers contributed to the person-year time between the date at the first of residency and the cancer diagnosis date. Meanwhile, those known to have died and who did not have cancers before the cutoff date contributed to the person-year time between their date of first residency and their date of death. A Cox proportional hazards model was used to assess the hazard ratio (HR) for cancer using the two radiation exposure assessments (distance from NPPs and cumulative dose of personal radiation) in the NPP cohort population, after adjusting for sex, year of birth, initial age of household registration, and medical radiation dose. This study only uses the internal and external exposure doses received by the radioactive gases and liquids discharged from the power plant to calculate the equivalent doses and effective doses; the background radiation value is not included in the final analysis. The baseline hazard functions is possible change during the long-term study period. Therefore, we will perform sensitivity analyses to stratify different ages and periods and limit the termination year to avoid long-term follow-up effect. The analysis was performed using SAS software (version 9.3; SAS Institute, Cary, NC, USA).

Additionally, this study uses a two-stage sampling approach to collect confounders and adjust for confounders in the final analysis.^27^ The estimated risk ratio of radiation exposure to health effects based on the “confirmed sample” established by the “two-stage sampling” method is then corrected for the estimated risk ratio of radiation exposure to health effects in the “non-confirmed sample”. According to Schaubel’s calculation by a balanced design, the estimated intensity (1-B) in the sample is set to 0.9; the odds ratio is set to 4.0 in the presence of an interference factor; prevalence of interference factor is 10%; q = 6 (q = p11p00/p10p01); 1,0 indicates whether there is an interference factor or whether it is an exposure group, and P is the prevalence rate. We estimate about 600 study samples were recruited, including near and non-near residents with and without cancers. Confounders, including personal habits, occupational history, medical history of radiation exposure, flying history, and pesticide exposure, were collected using a questionnaire. Adjusted risk ratios and hazard ratios will be used to express the net cancer risk of living near NPP after adjusting for confounders.

## DISCUSSION

The household population who had lived between 1978 and 2015 within 8 km of any of the three NPPs was regarded as the residence-nearest NPP group, with 214,502 people (4,697,593 person-years). In addition, 257,475 people (6,282,390 person-years) were selected as the control group, with similar geographic areas and population characteristics as the residence-nearest group but who had lived at least 15 km away from the NPPs. The two methods of radiation-exposure assessment adopt (1) the distance between the NPPs and the residence and (2) the nuclear power plant dose assessment. The dose of radioactive nuclear species routinely discharged from the three NPPs after the official operation from 1978 to 2015 was used to calculate the externally released dose. Then we established an annual equivalent dose and effective dose database base on the actual consumption of residents and the amount of residents engaged in specific operations. Based on each resident’s age group, the number of the NPP, his or her residence and distance from the NPP, the years of residence, and the proportion of residence in those years, this study calculated the cumulative effective dose to correspond to all cancer and 18 types of organ equivalent doses to 29 target cancers for each resident before cancer diagnosis. Considering the incubation periods of leukemia and solid cancer are 2 and 10 years, respectively, this study presents the number of new cancer cases and prevalence in the residence-nearest NPP group and the control group in the 38-year research observation period (Table 5).

**Table 5.** Number of new cases and prevalence in residence-nearest NPP group and control group

Sites/Types	Residence-nearest NPP group (*n* = 214,502)		Control group (*n* = 257,475)	
	*cases*	*‰*	*cases*	*‰*
Overall Cancer	11,809	55.05	15,648	60.77
1. Malignant neoplasm of lymphatic and haemopoietic tissue				
Lymphocytic leukemia	83	0.39	99	0.38
Leukemia (excluded Chronic lymphocytic)	75	0.35	86	0.33
Lymphoma	118	0.55	178	0.69
Hodgkin’s lymphoma	6	0.03	15	0.06
Non-Hodgkin’s lymphoma	112	0.52	163	0.63
Multiple myeloma	25	0.12	25	0.10
2. Malignant neoplasm of lip, oral cavity and pharynx				
Oral	669	3.12	676	2.63
Oral cavity and pharynx	1,192	5.56	1,312	5.10
Major salivary glands	39	0.18	44	0.17
3. Malignant neoplasm of digestive organs and peritoneum				
Esophagus	393	1.83	466	1.81
Stomach	811	3.78	1,024	3.98
Colon (excluding rectum)	841	3.92	1,224	4.75
Rectum and rectosigmoid junction, sigmoid colon, anus	707	3.30	855	3.32
Rectum and rectosigmoid junction	693	3.23	842	3.27
Sigmoid colon	321	1.50	487	1.89
Anus, anal canal, and anorectum	16	0.07	15	0.06
Liver and intrahepatic bile duct	1,301	6.07	2,132	8.28
4. Malignant neoplasm of respiratory and intrathoracic organs				
Lung	1,356	6.32	1,877	7.29
Trachea and bronchi	35	0.16	48	0.19
5. Malignant neoplasm of bone, connective tissue, skin and breast				
Bones, joints, and articular cartilage	35	0.16	44	0.17
Connective, subcutaneous and other soft tissues	67	0.31	100	0.39
Melanoma of skin	#		#	
Other non-melanoma skin cancer, excluding basal and squamous cell carcinoma	323	1.51	502	1.95
Female breast^a^	1,289	6.01	1,610	6.25
6. Malignant Neoplasm of Genital Organs				
Uterus, not otherwise specified^a^	5	0.02	5	0.02
Ovary, fallopian tube, and broad ligament^a^	159	0.74	256	0.99
Prostate gland^b^	400	1.86	459	1.78
7. Malignant neoplasms of urinary tract				
Bladder	272	1.27	506	1.97
Kidney	138	0.64	198	0.77
8. Malignant neoplasm of nervous system				
Brain	107	0.50	143	0.56
Other and unspecified parts of nervous system	7	0.03	7	0.03
9. Thyroid gland	341	1.59	453	1.76

NPP, Nuclear Power Plant.

^a^Analyzed only female residents.

^b^Analyzed only male residents.

### Medical radiation exposure

In order to avoid the influence of medical radiation exposure, this study linked to the National Health Insurance Research Database to get records of individual medical examinations, including conventional radiographic examinations, conventional fluoroscopic examinations, cardiac interventional radiology, non-cardiac interventional radiology and fluoroscopy, dental radiographs, computerized tomography, and nuclear medicine (Table 6). Moreover, we used every record from medical examinations to assess the cumulative medical radiation exposure dose (mSv) in each person, based on the population dose from medical exposure in Taiwan for 2008^28^ (eTable 3). Excluded medical uses of radiation categories and doses after a cancer diagnosis, the mean of cumulative medical radiation exposure dose between the residence-nearest NPP group and control group were not different (7.69; SD, 18.39 mSv and 7.61; SD, 19.17 mSv; *P* = 0.114).

**Table 6.** Cumulative medical uses of radiation categories and doses in residence-nearest NPP group and control group^a^

	Residence-nearest NPP group (*n* = 214,502)		Control group (*n* = 257,475)		
Medical examinations	*mean*	*SD*	*mean*	*SD*	*P-value*
Conventional radiographic procedures (times)	11.84	17.59	11.96	17.44	0.019
Conventional fluoroscopic procedures (times)	0.033	0.252	0.028	0.226	<0.001
Cardiac interventional fluoroscopic procedures (times)	0.071	0.628	0.074	0.643	0.067
Non-cardiac interventional radiology and fluoroscopy (times)	0.068	0.599	0.070	0.665	0.239
Dental radiographic procedures (times)	1.146	2.244	1.038	2.193	<0.001
Computed tomography (times)	0.637	1.532	0.632	1.498	0.304
Nuclear medicine (times)	0.080	0.441	0.078	0.428	0.088

Medical exposure dose					
Cumulative medical radiation exposure dose (mSv)	7.69	18.39	7.61	19.17	0.114

NPP, Nuclear Power Plant.

^a^This study excluded medical uses of radiation categories and doses after a cancer diagnosis.

### Strengths and limitations

This is the first multi-decade retrospective cohort study of residents around NPPs to present the health effects of extremely low radiation exposure doses during normal operation of NPPs. It drew upon detailed, dynamic personal household registration information to assess individual radiation exposure from 1978 to 2015. Moreover, it identified new cancer cases and information about cancer among the cohort and control population from TCR during the 38-year follow up. The TCR was established in 1979 to monitor the incidence and the mortality rates of cancer in Taiwan. Under the current system, the TCR captures 97% of the cancer cases in Taiwan.^29^ The quality of a cancer registry is indicated by the percentage of death certificate only cases (DCO%) and the percentage of morphologically verified cases (MV%), with the perfect data quality represented by a DCO% of 0 and a MV% of 100.^30^ The DCO% of the cancer cases in the TCR decreased from 8.78% in 1998 to 0.85% in 2010.^29^ The MV% ranged from 87.5% in 2002 to 91.11% in 2010.^29^ These indices show that the quality of the TCR is comparable to other well-established cancer registries in the world.^30^^,^^31^ Finally, the amount of radioactive materials released from NPPs have decreased year by year. This study can provide an accurate estimation using cumulative dose considering the length of residence, not just a single point in time, and provide integrated data of the residents near multiple NPPs for further analysis.

There are still some limitations in this study. The radiation dose is estimated under the assumption that all subjects have been at their registered residence all day because the information of commute to workplace or school and type of building is unavailable, and therefore estimated radiation dose has uncertainty. However, for the purpose of the reconstruction of the 38-year historical radiation exposure dose and to be estimated on the entire population, this could be the most satisfactory and feasible method. It employed household-registration data to establish our research cohort as a conservative estimate; because household registration may differ from actual place of residence, this may cause bias in the research results. We assume that under the normal operation of nuclear power plants, low-dose radiation will increase the cancer risk of surrounding residents. Therefore, if the actual occupancy rate is lower, the results of the research will be underestimated. Moreover, existing residents do not include those who have died or migrate due to illness, so the existing residents’ cohort is not used in this study. Based on the sample size estimation in eTable 4, if the Excess relative risk (ERR) of all-caused cancer is setting as 3%, the estimated number of person-years is 4,407,926. The sample size in this study that would afford this degree. However, if the ERR is setting as 2% or less, the estimated number of person-years must reach 9,869,177 or more. The larger sample sizes will be needed for the research to reach statistical significance. Although the truly cancer risk of exposed to chronic cumulated low-dose ionizing radiation has not been understood, this study could be difficult to collect this big sample sizes within a limited distance near NPPs.

### Conclusion

The TNPECS provides a valuable platform for research, opening unique possibilities for testing whether long-term radiation exposure from nuclear reactors affects health across the life course. Findings will provide evidence of cancer risk of ionizing radiation for government, in order to address a wide range of environmental and health concerns.

## SUPPLEMENTARY MATERIAL

eTable 1. Age at first residency for each period

eTable 2. International organizations identified or mentioned cancer sites related to ionizing radiation

eTable 3. The estimated average effective dose per radiographic procedure performed in Taiwan in 2008 for each procedure

eTable 4. Sample size estimation in this cohort study design
