# Supplementary material for: Study Protocol for Radiation Exposure and Cancer Risk Assessment: The Taiwan Nuclear Power Plants and Epidemiology Cohort Study (TNPECS)
Source: J Epidemiol. 2023 Jan 5;33(1):52–61. doi: 10.2188/jea.JE20210020 (PMC9727215; doi:10.2188/jea.JE20210020)
Supplement: Supplementary file 1 [file je-33-052-s001.pdf]

eTable 1. Age at first residency for each period

Age in the first year of residency	Residence-nearest NPP1 population			Residence-nearest NPP2 population			Residence-nearest NPP3 population			North control group			South control group		
Initial residency period	*n*	*mean*	*SD*	*n*	*mean*	*SD*	*n*	*mean*	*SD*	*n*	*mean*	*SD*	*n*	*mean*	*SD*
1978–1982	17,754	22.2	20.4	39,565	21.6	20.3				45,046	22.2	18.5			
1983–1987	20,210	24.8	20.9	49,667	24.4	21.0	29,565	23.9	19.6	49,537	24.8	19.3	34,752	23.9	19.5
1988–2000	27,689	24.9	22.3	84,302	22.9	22.0	38,963	23.4	21.4	71,585	23.0	21.5	45,835	23.4	21.4
2001–2015	26,142	26.7	21.3	98,496	26.7	21.3	54,533	29.4	22.9	80,072	26.5	21.2	63,876	29.6	22.8

eTable 2. International organizations identified or mentioned cancer sites related to ionizing radiation

	Published year						
**Sites/Types**	**IARC**	**ICRP**	**NAS**	**NCI**	**UNSCEAR**	**ERR$_{1000mSv}$ [a]**	**90%CI**
Overall Cancer	2012		1990	2011	2006	0.62	0.55–0.69
1. Malignant neoplasm of lymphatic and haemopoietic tissue							
Lymphocytic leukemia	1990, 2012	2007	1990	2011	2000, 2006	4.37	3.20–5.60
Hodgkin's lymphoma	1990		1990	2011	2006	0.25	<0.20–1.11
Non-Hodgkin's lymphoma	1990		1990	2011	2006	0.43	-1.60–3.50
Multiple myeloma	1990	2007	1990	2011	2006	0.20	<-0.20–1.70
2. Malignant neoplasm of lip, oral cavity and pharynx							
Oral	2012	2007				0.16	-0.06–0.50
Major salivary glands	2012	2007			2006	2.55	0.87–5.72
3. Malignant neoplasm of digestive organs and peritoneum							
Esophagus	2012	2007			2006	0.37	-0.45–1.31
Stomach	1990	2007	1990	2011	2000, 2006	0.37	0.26–0.49
Rectum and rectosigmoid junction, sigmoid colon, anus	1990; 2011	2007	1990	2011	2000; 2006	0.64 (Colon) 0.18 (Rectum)	0.42–0.90 <0–0.46
Liver and intrahepatic bile duct	1990	2007	1990	2011	2006	0.41	0.22–0.63
4. Malignant neoplasm of respiratory and intrathoracic organs							
Lung	1990; 2012	2007	1990	2011	2000	1.00	0.60–1.40
5. Malignant neoplasm of bone, connective tissue, skin and breast							
Bones, joints, and articular cartilage	2012	2007	1990	2011	2006		
Connective, subcutaneous and other soft tissues	2007				2000; 2006	1.42	<-0.2–4.5
Melanoma of skin	2012	2007			2000; 2006	0.88	0.40–1.90
Female breast	1990; 2012	2007	1990	2011	2000; 2006	1.49	1.17–1.85
6. Malignant Neoplasm of Genital Organs							
Ovary, fallopian tube, and broad ligament	2012	2007			2006	0.61	0.08–1.35 <0–0.32

						(Ovary)<br>0.10<br>(Uterus)	
Prostate gland	1990; 2012				2006	0.12	<0–0.51
7. Malignant neoplasms of urinary tract							
Bladder	1990; 2012	2007	1990	2011	2000; 2006	0.92	0.46–1.50
Kidney	1990; 2012				2006	0.16	<0–0.78
8. Malignant neoplasm of nervous system							
Brain and unspecified parts of nervous system	2012	2007	1990	2011	2006	0.22	<0–1.30
9. Thyroid	1990; 2012	2007	1990	2011	2000; 2006	1.59	1.10–2.19

[*] IARC, International Agency for Research on Cancer; ICRP, International Commission on Radiological Protection; NAS, United States National Academy of Sciences; NCI, National Cancer Institute; UNSCEAR, United Nations Scientific Committee on the Effects of Atomic Radiation; $ERR_{1000mSv}$, Excess Relative Risk/1,000 mSv.

[a] Data source from Preston et al., 1994, 2007 and Thompson et al., 1994

eTable 3. The estimated average effective dose per radiographic procedure performed in Taiwan in 2008 for each procedure

**Medical examinations**	**Body part or organ system**	**Average effective dose per procedure (mSv)(E_{103})** [a]
Conventional radiographic procedures	Chest	0.06
Conventional radiographic procedures	kidney, ureter, and bladder	0.38
Conventional radiographic procedures	Lower limb	0.002
Conventional radiographic procedures	Spine	1.15
Conventional radiographic procedures	Upper limb	0.001
Conventional radiographic procedures	Skull	0.04
Conventional radiographic procedures	Pelvis	0.44
Conventional radiographic procedures	Shoulder	0.07
Conventional radiographic procedures	Abdomen	0.48
Conventional fluoroscopic procedures	Intravenous Urogram	2.1
Conventional fluoroscopic procedures	Lower gastrointestinal (GI) series	3.5
Conventional fluoroscopic procedures	Upper GI series	4.5
Conventional fluoroscopic procedures	Esophagography	1.2
Conventional fluoroscopic procedures	Small bowel series	1.3
Conventional fluoroscopic procedures	T-tube cholecystography	1.1
Conventional fluoroscopic procedures	Retrograde pyelography-one side	1.55
Conventional fluoroscopic procedures	Antegrade pyelography	1.55
Conventional fluoroscopic procedures	Swallowing video fluorography	0.52
Conventional fluoroscopic procedures	Cystography	0.53
Conventional fluoroscopic procedures	Fistulography	2.65

Conventional fluoroscopic procedures	Retrograde pyelography-both sides	3.1
Cardiac interventional fluoroscopic procedures	Cardiac cath - one side	3.90
Cardiac interventional fluoroscopic procedures	Coronary angiography	3.9
Cardiac interventional fluoroscopic procedures	Cardioangiography	3.90
Cardiac interventional fluoroscopic procedures	Percutaneous coronary dilatation - one vessel	7.2
Cardiac interventional fluoroscopic procedures	Percutaneous coronary dilatation - two vessels	18.2
Cardiac interventional fluoroscopic procedures	Cardiac cath- both side	7.80
Cardiac interventional fluoroscopic procedures	Transcatheter radiofrequency	6.90
Noncardiac interventional fluoroscopic procedures	percutaneous transluminal angiography	23.0
Noncardiac interventional fluoroscopic procedures	transarterial embolization	32.3
Noncardiac interventional fluoroscopic procedures	Hysterosalpingography	0.6
Noncardiac interventional fluoroscopic procedures	Percutaneous transhepatic cholangiography drainage	1.2
Noncardiac interventional fluoroscopic procedures	Percutaneous nephrostomy	2.09
Noncardiac interventional fluoroscopic procedures	Voiding urethrocystography	1.86
Noncardiac interventional fluoroscopic procedures	Endoscopic retrograde cholangiopancreatography	5.0
Noncardiac interventional fluoroscopic procedures	CT-Guide biopsy	2.60
Noncardiac interventional fluoroscopic procedures	Arteriography of extremity	2.30
Noncardiac interventional fluoroscopic procedures	Percutaneous vertebroplasty	2.57
Noncardiac interventional fluoroscopic procedures	Visceral angiography	8.51
Noncardiac interventional fluoroscopic procedures	Aortography (cineangiography)	5.10
Noncardiac interventional fluoroscopic procedures	Common carotid artery-both side	8.45

Noncardiac interventional fluoroscopic procedures	Vertebral angiography	2.57
Noncardiac interventional fluoroscopic procedures	Arthrography	0.10
Noncardiac interventional fluoroscopic procedures	Antegrade venography	0.56
Noncardiac interventional fluoroscopic procedures	Lumber spine myelography	2.38
Noncardiac interventional fluoroscopic procedures	Intra aorta balloon insertion	5.10
Noncardiac interventional fluoroscopic procedures	Intravenous Digital Subtraction Angiography	1.30
Noncardiac interventional fluoroscopic procedures	Percutaneous gall bladder drainage	6.85
Noncardiac interventional fluoroscopic procedures	Subclavian angiography	3.60
Noncardiac interventional fluoroscopic procedures	Mammography sterotactic biopsy	2.60
Noncardiac interventional fluoroscopic procedures	Pulmonary angiography	8.20
Dental radiographic procedures	Periapical radiography	0.007
Dental radiographic procedures	Panoramic radiography	0.018
Dental radiographic procedures	Bite-wing radiography	0.011
Dental radiographic procedures	Temporomandibular joint radiography, unilateral	0.013
Dental radiographic procedures	Operculectomy	0.018
Dental radiographic procedures	Occlusal radiography	0.015
Dental radiographic procedures	Cephalometric radiography	0.009
Computed tomography	Head	1.8
Computed tomography	Chest	10.1
Computed tomography	Abdomen	7.5
Computed tomography	Pelvis	5.7
Nuclear Medicine	Whole body bone scan	4.44

Nuclear Medicine	Stress and redistribution myocardial perfusion study with SPECT	10.74
Nuclear Medicine	Positron emission tomography/CT	6.66
Nuclear Medicine	Ventricular ejection fraction measurement & wall motion study	3.70
Nuclear Medicine	Whole body inflammation scan	11.32
Nuclear Medicine	Dynamic renal/urinary function study	0.89
Nuclear Medicine	99mTc thyroid scan	2.22
Nuclear Medicine	Sialoscintigraphy	4.44
Nuclear Medicine	I-131 cancer work-up	11.47
Nuclear Medicine	Whole body tumor scan	18.87
Nuclear Medicine	Renal scan	0.89
Nuclear Medicine	Resting cardiac function study	10.74
Nuclear Medicine	Cerebral perfusion scan with SPECT	6.66
Nuclear Medicine	Three-phase bone scan	4.44
Nuclear Medicine	Lung perfusion scan	3.26
Nuclear Medicine	Lymphoscintogram	0.74

CT, computed tomography; SPECT, single photo emission CT.

[a] Based on the population dose from medical exposure in Taiwan for 2008[30]

eTable 4. Sample size estimation in this cohort study design

Cancer types (incidence rate)	RR=1.02 (ERR=2%)	RR=1.03 (ERR=3%)	RR=1.05 (ERR=5%)	RR=1.10 (ERR=10%)	RR=1.20 (ERR=20%)
	person-years	***person-years***	***person-years***	***person-years***	***person-years***
All-cause cancers (400/100,000)	9,869,177	4,407,926	1,602,423	410,335	53,905
Solid tumors (382/100,000)	10,336,103	4,616,476	1,678,240	429,752	56,445

ERR, excessive relative risk; RR, relative risk.
